# Crisis upon crisis: a qualitative study exploring the joint effect of the political, economic, and pandemic challenges in Lebanon on Syrian refugee women’s fertility preferences and behaviour

**DOI:** 10.1186/s13031-022-00468-8

**Published:** 2022-06-15

**Authors:** Rima Mourtada, Andrea J. Melnikas

**Affiliations:** 1 Beirut, Lebanon; 2 Population Council, One Dag Hammarskjold Plaza, New York, NY 10017 USA

**Keywords:** Refugees, Crisis, Covid-19, Pandemic, Fertility preferences, Fertility practices, Family Planning, Contraception

## Abstract

**Background:**

Starting in October 2019, Lebanon experienced overlapping crises that caused a significant deterioration of the living conditions for Syrian refugees and the host community. Previous studies have shown that difficult living conditions and refugee status alone do not impact the fertility preferences of Syrian refugees. This study seeks to explore the effect of the overlapping crises on the fertility preferences and behaviour of Syrian refugees in Lebanon.

**Methods:**

In this qualitative study, we carried out focus group discussions (FGDs) with married female Syrian refugees recruited purposively from two cities in West Bekaa (Bar Elias and Saad Nayel) and from inside and outside the Informal Tented Settlements (ITS). Transcripts were analysed using thematic analysis.

**Results:**

The overlapping crises (political, economic, and Covid-19) in Lebanon influence Syrian refugee women’s reported desire for fewer children. Two themes emerged that explained the change in Syrian refugees’ fertility preferences towards limiting their number of children or delaying having children, and potentially a change in their fertility practices: the sudden deterioration in their living conditions triggered by the effect of inflation on their daily needs, and decreased support and changes in the job market that led to more women working to support their families. Consequently, refugees expressed a preference towards limiting their number of children due to concern about the consequences of the crisis on their children’s physical and mental well-being. This was combined with decreased pressure on women from men and in-laws to have (additional) children and concern over the effect of Covid-19 on pregnant women.

**Conclusions:**

The sudden deterioration in living conditions due to the overlapping crises may have influenced Syrian refugees’ preferences towards limiting their number of children or delaying having children until the situation improves. The potential shift in power dynamics in households caused by more women working outside the home also may have increased women’s autonomy in making decisions regarding family size and use of modern contraception. These findings have implications for developing programs that focus on female livelihoods and engagement in work outside the home to influence family size and other reproductive health outcomes and gender equity indicators.

## Background

Since the start of the Syrian conflict almost a decade ago, Lebanon has been hosting the largest number of Syrian refugees per capita. According to the United Nations High Commissioner for Refugees (UNHCR), there are currently around 855,000 registered Syrian refugees in Lebanon [1]. Since their resettlement in Lebanon, Syrian refugees have faced many challenges, such as limited work opportunities, poor living conditions, tensions with the host community, and reduced access to essential services like health, education, and legal services, especially marriage and birth registrations [2, 3]. The latest vulnerability assessment of Syrian refugees (VASYR) in Lebanon in 2020 indicated that 67% of Syrian refugees in Lebanon live in residential structures, 21% live in informal tented settlements (ITS), and around 12% live in non-residential units, as unlike other neighbouring countries, Lebanon did not establish formal refugee camps [4]. The same assessment also revealed that almost 80% of refugees do not have a legal residency permit, which therefore limits their access to basic services and exposes them to additional risks of exploitation and prosecution [4]. The most economically disadvantaged refugees are supported by UNHCR and the World Food Program (WFP) through various cash assistance mechanisms. Additionally, UNHCR subsidizes primary health centre fees and covers up to 90% of hospital fees for deliveries and life-threatening conditions. However, unregistered refugees (estimated at over 500,000) are excluded from such assistance mechanisms [5].

Health services, including reproductive health services (RHS), provided for refugees are generally designed by humanitarian groups as temporary interventions that address the refugees’ immediate needs. These services often aim to address acute health needs, such as nutrition, and focus less on what may be perceived as long-term needs such as family planning (FP), which is essential for refugees who end up displaced for years [6], such as Syrian refugees in Lebanon. Extreme changes in life circumstances are likely to influence fertility preferences [6]. When it comes to refugees/migrants’ fertility preferences and practices, there is mixed evidence about the effect of displacement on fertility behaviour post migration/displacement [7]. As in many dynamic settings, refugee women’s fertility preferences may shift, causing a change in their contraceptive needs, which may be different from the local population or even different from their own community of origin [8, 9]. It is difficult to predict long-term fertility behaviour in forced migration settings as fertility behaviour is influenced by numerous and interrelated factors [10].

Despite the decline in total fertility rate (TFR) in Syria, which dropped from 5.3 in 1990 to 2.9 in 2010, Syria had the sixth highest TFR in the Arab World in 2010 [11]. There are no reliable quantitative figures on TFR among Syrians within Syria or among Syrian refugees in Lebanon after the conflict, however the proportion of women whose demand for FP was met declined considerably after the conflict, especially among Syrian refugees in Lebanon [12].

A few nonrepresentative qualitative studies suggest that Syrian refugees maintained their pre-conflict preferences for large family size and reported limited use of modern contraception methods [3, 13–15]. Reported reasons for family size preservation were traditions, religion, and low agency among women that persisted post-displacement [3]. One of the studies reported that many refugees were not in favour of using modern contraception methods [3]. Although some of the reasons for not using modern contraception methods were related to service provision and the available contraception methods that did not meet women’s needs and expectations [3], refugees reported additional reasons for desiring a large family that were specifically related to war and displacement. Those reasons included replacing lost children who were killed in war, the additional help that larger families received from UNHCR (especially at the early stages of the displacement), and the ability of young children to work in agriculture and support their families, which was particularly important given the paucity of economic opportunities available to Syrian refugees [3]. The same study demonstrated that men were often the main decision makers regarding family size and the use of contraception. Women reported concerns about their husbands marrying another woman, which was a common practice, and thus submitted to men’s preferences for a larger family size [3]. Other studies in Lebanon demonstrated similar findings in relation to Syrian refugee women’s low agency in the decision-making process regarding the desired number of children and use of modern contraception methods [13–15].

A review paper on forced migration and fertility discussed that prolonged social and political exclusion may sometimes encourage or sustain high fertility in refugee camps despite poor economic conditions that would be expected to reduce fertility [16]. A possible explanation of the high fertility is their limited access to RHS due to their isolation. Another potential explanation is the exclusion of refugees, which may discourage them from adjusting their reproductive preferences [16].

Despite the various challenges that Syrian refugees faced since their displacement to Lebanon, post-displacement life circumstances did not seem to trigger a major change in their pre-conflict attitudes towards the ideal family size and use of modern contraception [3]. However, Syrian refugees’ attitudes may have changed given the recent financial, political, and pandemic crises in Lebanon since 2019.

The elevated levels of public-sector debt in Lebanon, in addition to the bankruptcy of the banking sector due to lending three-quarters of deposits to the government and the lack of growth in the productive economy resulted in a sudden stop in capital flows and a financial crisis in October 2019 [17]. This triggered systemic failures across the banking debt sectors, consequently affecting the exchange rate and resulting in inflation [17]. Given the financial crisis, the Lebanese government decided to impose a new tax on WhatsApp voice calls, which in turn led to political unrest that was manifested by protests and roadblocks in different locations in the country in an attempt to end the corruption and force all political parties to resign [18]. The financial and political crises were soon aggravated by the Lebanese government’s measures to control the transmission of COVID-19, which comprised the closure of the borders (air, sea, and land) and public and private institutions, and a number of prolonged periods of national lockdowns between March 2020 and March 2021 [18]. In 2020, the annual inflation rate reached 84.9% compared to only 2.9% in 2019 and consumer prices jumped. For instance, in 2020, food prices rose 402.3% from 2019 [19]. Since October 2019, The Lebanese Pound (LBP) lost at least 85% of its value [20]. The crises caused a worsening of living conditions for both Lebanese nationals and refugees due to the loss of jobs and reduced support [21]. The reported unemployment rate among refugees rose from 31% in 2019 to 39% in 2020, and more women were employed (45%) than men (38%) [4]. The latest VASYR assessment reported that because of the multiple crises, almost 89% of the refugee population lived below the Survival and Minimum Expenditure Baskets (SMEB),^1^ a huge increase from the 55% figure from the previous year [4]. Additionally, the crises caused a tremendous increase in the number of Syrian refugee households that had debt [4]. Moreover, support from assistance programs, the main source of support for many refugees, was no longer sufficient due to the multiple exchange dollar rates (at least four different rates) that fluctuated daily and caused a considerable decrease in the value of assistance by humanitarian organizations [20].

Given the multiple crises, we anticipated that the extreme and sudden change in refugees’ life circumstances resulting from the joint effect of the political, economic, and pandemic crises likely caused a shift in Syrian refugees’ fertility preferences towards limiting their number of children or delaying having children and could have resulted in an increase in their demand for FP and modern contraception. However, the potential disruption in the provision of RHS and modern contraception for Syrian refugee women due to Covid-19, which was reported in other nonhumanitarian settings [22] could result in an increase in the unmet need for FP among Syrian refugees in Lebanon. Increases in unmet need for FP are associated with negative health outcomes including unwanted pregnancies and unsafe abortion [23]. Moreover, high fertility in contexts that limit access to basic services such as marriage and birth registration may have dire implications and result in an increased number of stateless children with no access to education and health. Understanding how Syrian refugees’ fertility preferences changed in light of the current circumstances may contribute to helping service providers meet women’s contraception needs.

## Methods

### Data collection

**Fig. 1:**
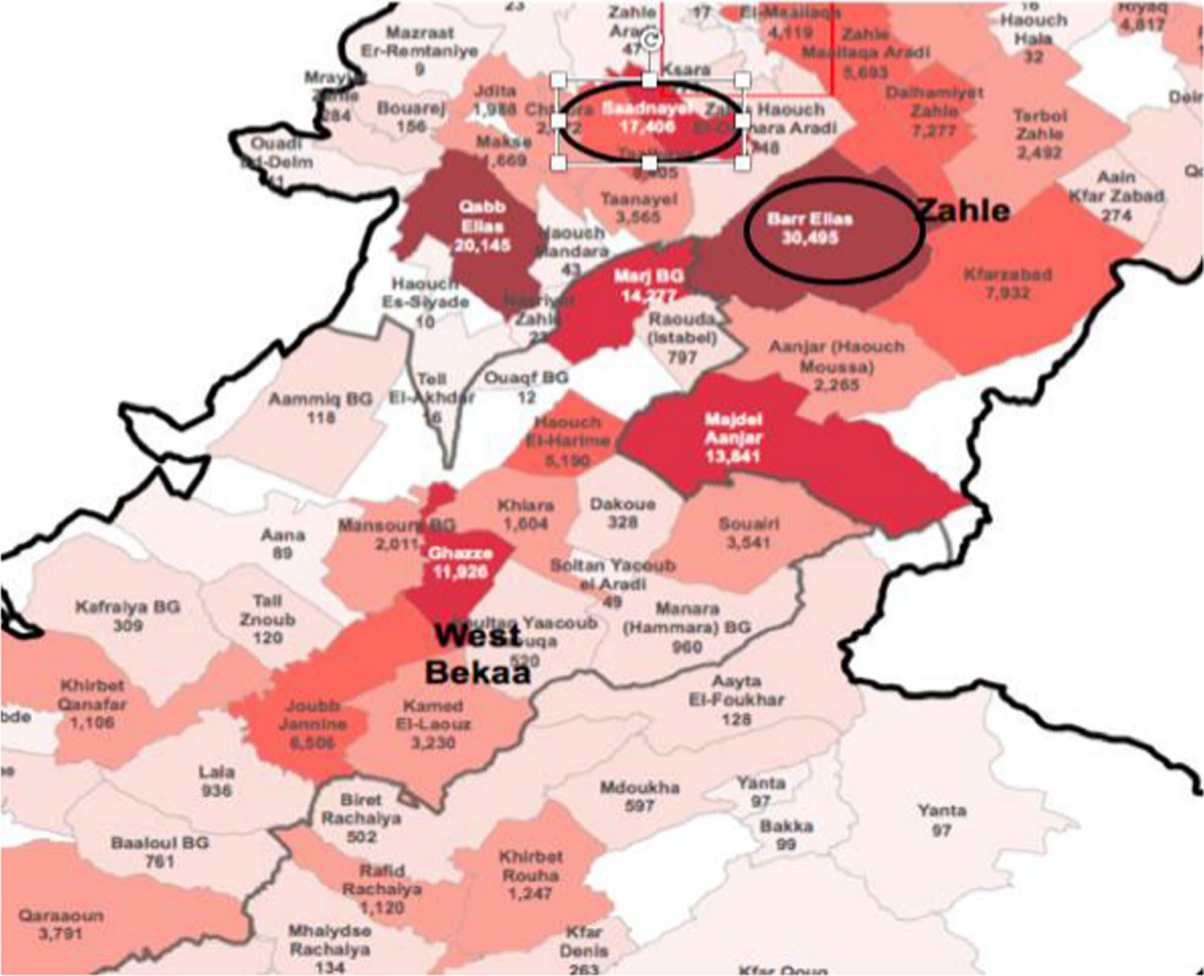
A snapshot of West Bekaa including the towns from which study participants were selected ( *Source* UNHCR data portal)

This study was part of a larger exploratory qualitative study that aimed to investigate how demand for and access to RHS and modern contraception methods were influenced by the political, economic, and pandemic crises in Lebanon. The study was carried out in two cities in West Bekaa that host a large number of Syrian refugees: Bar Elias and Saad Nayel (Fig. 1).

### Eligible participants

We selected three age groups to include women at different reproductive life stages (young married women ages 15–19; older married women ages 20–35; and mothers and mothers-in-law of young married women) anticipating that different age groups would reflect diverse fertility preferences and behaviour. The youngest age group represented women who either did not have children yet or who did not complete their desired family size. We also included older women because they have influence on younger women’s reproductive health choices and use of FP.

We favoured using a qualitative study rather than using a survey because we were mainly interested in understanding the different pathways through which the recent crises may have impacted women’s fertility preferences and behaviours and these are not easily captured in quantitative studies. We employed FGDs because they permit an interpersonal exchange of ideas, which is not only encouraging to participants but allows us to compare the points of view exchanged by different participants, which is not possible when using individual interviews [24, 25].

### Recruitment of participants

In each of the selected cities, two trained community workers identified one large ITS to ensure the availability of the required number of eligible participants. To recruit women within the ITS, community workers met with eligible women in each of those settlements, explained the study, and documented the names and phone numbers of those who agreed to participate. To recruit women outside the ITS, community workers used the help of their colleagues and social circle to connect them with eligible women who lived outside the ITS in each of those cities. They contacted all identified eligible women using WhatsApp (which is commonly used in those communities), explained the study, and documented the names and phone numbers of those who agreed to participate. Women who agreed to participate were contacted later by the community workers to arrange for their participation in the FGD. Community workers were trained on basic ethical standards by the first author before the start of the study.

Data collection took place between July and October 2020. Due to Covid-19 and concerns about in-person data collection, FGDs were moderated by the first author using Skype with the help of trained community workers. The Skype interviews with women living inside the ITS were carried out at one of the big tents inside the selected ITS and FGDs with women living outside the ITS were carried out at a community centre that was easily accessible for women who lived in both selected cities. We booked the centre on days where there were no other activities taking place to prevent overcrowding and to comply with the governmental regulations regarding Covid-19. Masks and sanitizer were provided and respondents were seated apart from each other to maintain appropriate spacing.

### Instruments

The FGD guide covered the following topics: the current hardships experienced by refugees that emerged since the political, economic, and pandemic crises, and the influence of the crises on refugees’ perception of ideal number of children and their use of RHS and modern contraception methods. The guide was pretested before the start of the data collection.

### Data analyses

All interviews were conducted in Arabic, audio recorded, and transcribed verbatim by community workers. The author (RM) translated the transcribed interviews into English. Both authors (RM) and (AM) engaged in analysing the data using deductive thematic analysis as described by Braun and Clarke [26, 27]. The authors started by familiarizing themselves with the data and documenting and discussing their initial thoughts about potential themes. In the second step, the authors developed a coding manual that helped with organizing the data to support the interpretation. In the third step, the authors sorted the coded data into themes and subthemes. In the last two steps, the authors reviewed the themes and compared them against the raw data, then they defined and named the final themes.

## Results

A total of 119 women/girls participated in FGDs. Demographic characteristics of recruited participants are presented in Table 1.

**Table 1:** Demographic characteristics of women who participated in FGDs

N = 119			
	15–19 years old (n = 39)	20–35 years old (n = 40)	Mothers and mothers-in-law (n = 40)
Mean age	18	28	46
*Education n (%)*			
No school	4 (10)	1 (2)	9 (22.5)
Preparatory	21 (54)	18 (45)	16 (40)
Elementary	10 (26)	9 (23)	12 (30)
Secondary and above	4 (10)	12 (30)	3 (7.5)
*Age at marriage n (%)*			
12–15	25 (64)	6 (15)	11 (27.5)
16–17	12 (31)	15 (38)	12 (30)
18 and above	2 (5)	19 (47)	17 (42.5)
*Parity n (%)*			
No children	8 (21)	1 (2)	NA*
1	19 (49)	6 (15)	0
2–4	12 (31)	27 (68)	9 (22.5)
5 and above	0	6 (15)	31 (77.5)
*Age at first pregnancy (out of those who ever had children) n (%)*			
12–15	13 (42)	1 (3)	5 (12.5)
16–17	16 (52)	11 (28)	9 (22.5)
18–19	2 (6)	12 (31)	15 (37.5)
20 and above	NA	15 (38)	11 (27.5)

*NA Not applicable

We carried out 12 FGDs. In each city and for each age group, we conducted one FGD with women living inside the ITS and one FGD with women living outside the ITS.

### Themes

There was general agreement among participants about two major themes that explore how the joint crises caused a recent deterioration in the living conditions of Syrian refugees and how those may have contributed to shifting refugees’ fertility preferences towards limiting their number of children or delaying having children until after the crises (Table 2). Additional analyses that focused on reproductive health seeking behaviour and access to/use of modern contraception are presented elsewhere.

**Table 2:** Themes and subthemes

Themes	Subthemes
1-Deterioration of living conditions	Effect of inflation on daily needs Insufficient support for refugees Negative changes in the job market
2- Preferences towards limiting the number of children in the family	Concern about negative consequences of the crisis on children Decreased pressure from men and in-laws Concern regarding the consequences of Covid-19 on pregnant women Potential change in fertility practices post-crises

### Deterioration of living conditions

There was strong agreement among participants across all age categories and in both areas that Syrian refugees in Lebanon experienced a recent depreciation in their living conditions that was triggered by the political and economic crises. Although Syrian refugees endured many challenges since their displacement to Lebanon, it appears that the combined effect of the crises has exacerbated their challenges and resulted in further deterioration of their living conditions. Participants expressed concern over three major challenges that impacted their lives: the current inflation, insufficient material support received, and negative changes in the job market.

#### Effect of inflation on daily needs

The majority of women complained that the current inflation, caused by the economic crisis, was one of the main triggers behind the deterioration of their living conditions mainly because of the rapid increase in consumer goods prices.

“This year has been difficult. We used to be ok, but everything changed since the increase in the dollar value.” (20–35-year-old women inside the ITS, Saad Nayel)

As a way to cope with the economic crisis, women reported reprioritizing their needs and giving up on desired items in order to afford essential items.

“Now we deprived ourselves from many things, such as meat and vegetables. We get half of the amount we used to get. No one is able to afford everything with the increase in the prices.” (Mothers and mothers-in-law outside the ITS, Saad Nayel).

Because the COVID-19 virus was not widespread in the Bekaa area at the beginning of the pandemic, some women were less concerned about being infected with the virus and more concerned about the difficult living conditions and the consequences of the lockdown.

“Things changed since the demonstrations and since the increase in the dollar value. Corona did not affect us much, it only caused shops to close. Now we must cut our expenses. We eat less and we spend less.” (20–35-year-old women inside the ITS, Saad Nayel).

#### Insufficient material support for refugees

The United Nations distributes food vouchers to selected Syrian refugee families (based on certain vulnerability criteria). Although UNHCR increased the value of food vouchers since the pandemic to keep up with the current crisis, this increase was perceived as inadequate given the rapid surge of consumer goods prices.

“Two months ago, since Corona started, they increased the value of the food voucher to 40,000 LBP, then to 50,000 LBP last month and this month it became 60,000 LBP per person, but despite this increase, I went to the supermarket and I was not able to buy many things.” (20–35-year-old women outside the ITS, Saad Nayel).

Some respondents noted that because of the crises, host community members that had been welcoming and supportive to refugees were no longer able to provide that support. This was manifested mainly in two ways: their inability to offer loans to refugees and threats to evict refugees from their rented properties when they could not pay rent on time.

“No one agrees to give you loans and all people became poor. Everyone is dying out of hunger. The tragedy affected everyone equally.” (20–35-year-old women outside the ITS, Bar Elias).

“Landlords are no longer patient and it is their right as they also have responsibilities, but we are also suffering.” (20–35-year-old women outside the ITS, Saad Nayel)

#### Negative changes in the job market

The deterioration of the living conditions of Syrian refugees in Lebanon was caused in part by the disruption to an already restricted job market, brought on by Covid-19 lockdown regulations. All women agreed that one of the main challenges they were facing was men losing their jobs and the financial repercussions of the loss of income.

“Our husbands became jobless and are constantly at home; we no longer have a regular source of money.” (15–19-year-old women inside the ITS, Bar Elias)

The few refugees who managed to maintain their jobs throughout the crises complained about their current salaries that did not increase to keep up with the current economic crisis.

“Those who work are still receiving the same salaries. The salaries did not increase (all women in the group agreed).” (20–35-year-old women outside the ITS, Saad Nayel)

To cope with men losing their jobs and the difficult living conditions, an increasing number of women and children started to work in agriculture, which unlike other occupations was not as affected by the imposed lockdown. Generally, women and children are more likely to be employed in the agriculture sector because these jobs pay lower wages, which men would not accept. Additionally, employers prefer to hire women and children as they can easily control them as reported by participants in the FGDs.

“We had to send our children to work in agriculture (potatoes) because everyone [men] became jobless because of the crisis.” (Mothers and mothers-in-law inside the ITS, Bar Elias).

Even women living outside the ITS or those who had young children and who generally did not work before the crisis reported being obliged to work to be able to survive.

“Many women who are still breastfeeding are obliged to leave their young children to work and help with the house expenses.” (20–35-year-old women inside the ITS, Bar Elias)

Although Covid-19 was not a direct threat to refugees given its limited spread in the Bekaa area at the time of the study, participants were well aware of its negative implications on their daily lives resulting in limited mobility, decreased access to services, and the majority of men losing their jobs. This was concerning to them especially with the current inflation that resulted from the political and financial crises coupled with the decreased support from aid agencies and the local community. It appears that the magnified financial implications caused by the overlapping crises that hit the country at the same time may have caused a shift in refugees fertility preferences towards limiting the number of children in the family or delaying having children.

### Preferences towards limiting the number of children

The majority of participants across all FGDs voluntarily expressed their desire to limit the number of children in the family. The main reasons women reported behind their desire to limit the number of children in the family or delay having children as a result of the crisis included: increased stress and concern over the negative consequences of the crisis on children; decreased familial pressure to have children as women’s family role may have changed and paid work took precedence; concern over the health consequences of Covid-19 on pregnant women; and potential change in women’s fertility practices as a result of preferences to limit the number of children.

#### Increased stress and concern over negative consequences of the crisis on children

The negative consequences of the crises on children were brought up on many occasions. Participants described the negative consequences as children’s frustration due to their parents’ inability to provide for them, children’s inability to go to school due to school closures because of the government’s Covid-19 lockdown, and mothers’ concerns about the increase in violence within the household and its effect on their children.

Most women expressed frustration because of their inability to provide for their children, which often forced them to delay or stop having children given the current challenges. This was also expressed by women who belonged to the youngest age category and who did not achieve their desired family size yet.

“There is nothing more beautiful than children, that is if we can afford them, but it would be unfair to bring them to this world under the current circumstances.” (15–19-year-old women living inside the ITS, Saad Nayel).

Women especially complained about the increase in the prices of two essential items that young children need: diapers and milk.

“You need to think about providing milk and diapers, and going to the doctor, especially when most roads are closed. There are many things that discourage you from having children.” (15–19-year-old women outside the ITS, Bar Elias).

Road closures in response to demonstrations that began in October 2019 and Covid-19- related lockdowns resulted in school closures. Consequently, children were forced to stay home, which was reported as a main concern for many mothers.

“Our children used to go to school, they used to attend English courses during summer. They are no longer able to do that.” (20–35-year-old women outside the ITS, Bar Elias)

Some women reported that the current challenges increased the stress level within the household, especially among men who often became jobless. Some participants noted that increased stress resulted in men becoming more violent and taking their stress out on their family members, including children.

“My husband is constantly stressed out because he lost his job, and he beats our four-year-old whenever he plays. He takes his stress out on him. He never did this before. He even argues with his own father.” (15–19-year-old women living outside the ITS, Saad Nayel).

##### Decreased pressure from men and in-laws

The pressure to have children that used to be imposed by husbands and in-laws before the crisis seems to have diminished due to the current difficult conditions. Women reported that before the crisis, they were unable to voice their opinion about limiting the number of children they wanted to have out of fear that their husbands might obtain a second wife to have more children, which was a common practice before the crises. The crises that resulted in men losing their jobs seemed to affect this practice as most women indicated. The crises also influenced women’s role in families as more women reported taking paid work outside the home.

Many women expressed relief that their husbands’ decreased financial status made them unable to obtain a second wife: *“Men used to get a second wife when they wanted more children. My husband left us and remarried but the new generation cannot afford it.“ (Mothers and mothers-in-law inside the ITS, Bar Elias).*

In one group, women joked saying that the financial situation has reduced men’s options for remarrying: *“Although the situation is bad, with the increase in the dollar value, men can no longer remarry as they became jobless (everyone laughs).” (20–35-year-old women inside the settlements).*

Struggles of not being able to afford expenses for pregnant women (including the expenses of antenatal care and childbirth) as well as the expenses of young children were also commonly reported by mothers and mothers-in-law. This was particularly true among those living within extended family households and who, in normal situations, would encourage their daughters and/or daughters-in-law to have more children. However, due to the current challenges, some mothers/mothers-in-law reported advising their children/daughters-in-law to limit their number of children.

“My daughter-in-law has two boys and a girl; the oldest is four years old and the youngest is one year old. I advised her to stop because of the difficult living situation. I took her to the centre to have an IUD inserted, with her and her husband’s approval of course.” (Mothers and mothers-in-law outside the ITS, Bar Elias).

On rare occasions where pressure from in-laws to have children was exhibited, daughters-in-law appeared to have higher autonomy and confidence in limiting the number of children they want to have, justified by the current difficult conditions and their inability to provide for their children.

“We did not consult with my in-laws. I decided with my husband that we need to stop because we can no longer provide for our three children.” (20–35-year-old women inside the ITS, Bar Elias).

#### Concern regarding being infected with Covid-19 while pregnant

Surprisingly, concerns over being infected with Covid-19 while pregnant and the associated consequences on mothers and babies were only brought up by 20–35-year-old women who lived outside the ITS in the Bar Elias area. A few women belonging to this group articulated their concern about becoming pregnant and getting infected with Covid-19, which discouraged them from having more children.

“To be honest, the internet is also very informative. We heard that because of Corona it is dangerous for a woman to get pregnant. She would be exposing herself to dangers if she decides to get pregnant. We really think twice about this issue now.” (20–35-year-old women outside the ITS, Bar Elias).

#### Potential change in women’s fertility practices

It appears that the difficult living conditions and their negative effects on children may have influenced women’s fertility practices as some reported that they started using modern contraception methods since the crises, which was a clear indication of refugees’ desire to limit their number of children or to delay having children until after the crisis.

“Those who used to think about having more children stopped thinking about it. We all had an IUD inserted (they all laugh).” (20–35-year-old women inside the ITS)

Reports of using or being willing to use modern contraception methods was also expressed by women who belonged to the youngest age group who in normal circumstances would not use them.

“I also use pills. I started using them one month ago. My children are young and I get pregnant easily, so I decided to use them.” (15–19-year-old women inside the ITS, Bar Elias)

Despite their desire to limit their number of children, younger women were sometimes discouraged, by both their families and health providers, from using modern contraception methods, which caused them additional frustration given the current challenges. One woman expressed her frustration:

“I have one daughter only so they told me that I should not have an IUD inserted. I went to the doctor because I had back pain. She told me you have been pregnant for 40 days and I was able to listen to his heartbeats. I started crying.” (15–19-year-old women inside the ITS, Bar Elias).

Despite that, some women who did not complete their desired family size reported using modern contraception. *“I have only one daughter, but I had an IUD inserted since last year.” (15–19-year-old women inside the ITS, Saad Nayel).*

Although women were not explicit about their recent ability to assert their opinions regarding the size of their family or about using a modern contraception method, it appears that the economic hardship caused by the crises may have increased their autonomy possibly by providing new avenues for agency and negotiating power deriving from the decreased financial status of their husbands and their ability to work and support their families financially.

## Discussion

This qualitative study demonstrated two major interrelated themes: the sudden deterioration in the living conditions of Syrian refugees, and a potential recent shift in women’s fertility preferences towards limiting their number of children or delaying having children, which consequently indicated a potential change in the fertility behaviour of some participants. Our findings differ from previous research, which attempted to understand the fertility behaviour of Syrian refugees in Lebanon, and which demonstrated that despite their difficult living conditions post-displacement, many refugees maintained pre-conflict preferences for having a large family [3]. Our findings suggest that a sudden change in life circumstances due to overlapping crises may have a different influence on fertility preferences. This sudden change potentially caused a shift in refugees’ fertility preferences towards limiting their number of children or postponing having children, perhaps through the pathway of increased agency and choice for women vis-à-vis their partners, which was absent before the Syrian conflict as well as before the crises in Lebanon. We believe this situation is distinct from other studies of fertility preferences and behaviours among refugees due to how these crises overlap and exacerbate each other.

**Fig. 2:**
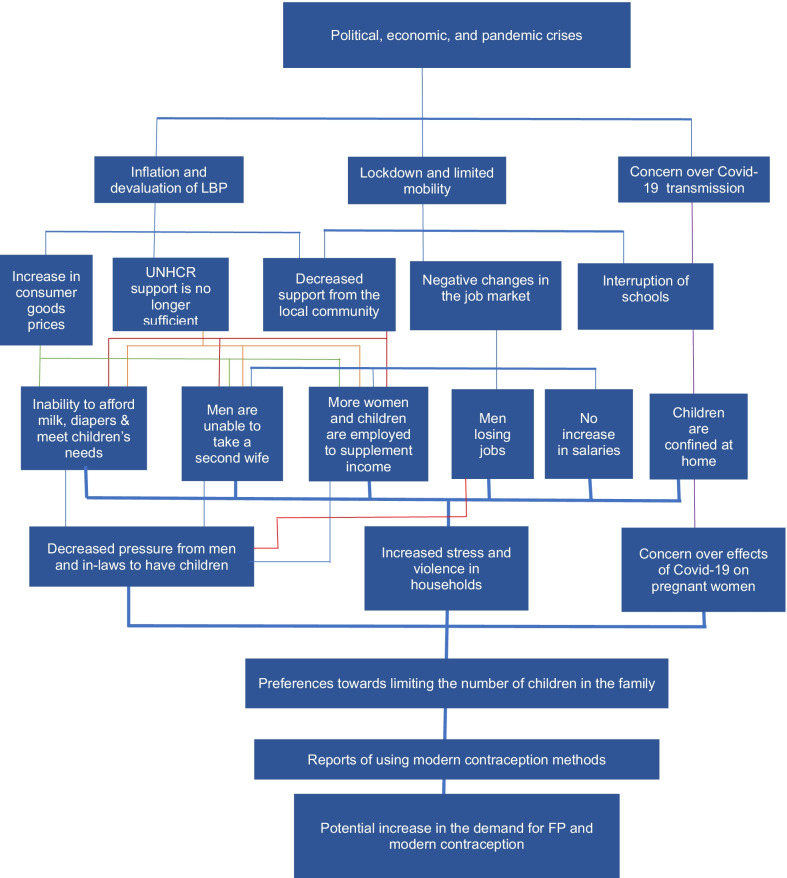
The joint effect of the crises on Syrian refugee women’s fertility preferences and behaviours

Based on the main findings, we highlight in Fig. 2 the different pathways through which the joint effect of the crises could have influenced women’s fertility preferences and consequently their behaviour.

As shown in Fig. 2, the political and economic crises, which caused rapid inflation and devaluation of the LBP, led to a considerable increase in consumer goods prices and a decrease in the value of food vouchers provided by UNHCR as well as support from the local community, as the crises affected everyone including the Lebanese nationals. Simultaneously, the imposed lockdown and limited mobility to control Covid-19 resulted in negative changes in the job market whereby most refugee men lost their jobs or did not earn enough to keep up with inflation. Consequently, men were no longer able to get a second wife (a common practice before the crises). At the same time, more women and children, who are usually employed in the agricultural sector for lower wages, started working to be able to cope with the difficult living conditions. Concurrently, the lockdown also interrupted schools and forced children to stay at home. Negative coping mechanisms among Syrian families were reported by the most recent vulnerability assessment of Syrian refugees in Lebanon. Some of these mechanisms included decreased food intake and increased child labour and child marriage [4].

The inability to afford basic needs, combined with the negative changes in the job market and children’s confinement at home may have jointly affected fertility intentions and behaviours possibly by decreasing the pressure exhibited (in normal circumstances) by men and in-laws on women to have more children. At the same time, the negative changes in the job market, which resulted in most men losing their jobs, the inability of men to take a second wife, and the increase in women’s employment, may have caused a shift in family power dynamics and could have resulted in either increasing women’s autonomy level and decision making regarding the desired family size and use of modern contraception or decreasing the adversity exhibited from men and in-laws when women expressed their desire to limit their number of children or both. Participants also reported concern over the psychological well-being of children and increased violence within households, which increased since the crises. A recent cross-sectional survey of 129 Syrian refugee families in Lebanon revealed that 83% of the surveyed families reported change in the children’s behaviour during the lockdown periods [21]. Stress and anxiety were the most frequent reported behavioural changes among both adults and children [21].

Our findings suggest that the economic hardships led to decreased pressure on women to have children. This combined with concerns about children’s well-being, and concern over the effect of Covid-19 on pregnancy outcomes, may have contributed to shifting refugees’ fertility preferences. As a result, some women reported using modern contraception methods since the crises, including younger women who did not complete their family size despite previous studies in Lebanon and other refugee settings that have demonstrated that the use of contraception is often not supported by husbands [3, 28].

The potential increase in demand for FP and the additional disruption of provision of and access to sexual and reproductive health services (SRHS) caused by the crises have important implications on service provision, especially in a country that already had an overburdened health system before the crises [29].

The Covid-19 situation differs across countries, and the roles of the public and private health sectors, available contraception methods, and supply chain barriers are also different [30]. Therefore, a country’s specific response will depend on how the pandemic unfolds as well as the choices women make regarding contraception use [30]. In order to address any potential unmet need in FP, there is a need to understand the current barriers to provision of and access to SRHS and modern contraception in Lebanon. Failing to address barriers in both provision of and access to services may result in severe and long term social and health implications.

The increase in female engagement in the workforce may partially explain women’s reported increase in autonomy, due to their increased economic importance in the household [31]. This increased stature within the household may enable women to make their own decisions regarding use of contraception, which could be the case for Syrian refugee women who started working as a result of men losing their jobs. This transformative nature of agency was also highlighted in an article describing the impact of internal displacement on women’s agency in two resettlement locations in Sri Lanka [32]. The article also emphasised the transformative nature of agency during resettlement and how women used it to reestablish their livelihoods in the resettlement areas [32]. These findings suggest that development programs that focus on female livelihoods and engagement in work outside the home may influence family size, other sexual and reproductive health (SRH) outcomes, and gender equity indicators as well.

## Study limitations

There are a few limitations to consider alongside the results from this study. The sample included in this study is not representative of all women in both cities or all Syrian refugee women, but since this is qualitative research, the sample is not selected for statistical representativeness, but rather to understand social processes under different conditions. Additionally, although FGDs allow an exchange of ideas between participants, there is a chance that some women may have been reluctant to share their opinion if they felt that other women in the group would not endorse it. Due to the pandemic, the community workers had little control over the recruitment method and counted on WhatsApp to invite eligible women to participate in the FGD, therefore it was not always easy to ensure that women in the group did not know each other.

An additional limitation is the absence of men in this study. Interviewing men about their attitudes regarding fertility as well as their views on using modern contraception given the current crises would have enriched and strengthened our findings. Moderating FGDs with men about such sensitive topics and in this culture could only have been feasible if the discussions were moderated by a male researcher. Unfortunately, time, pandemic, and budget limitations did not permit arranging for such interviews.

Although face-to-face interviewing in qualitative research is considered the gold standard, the use of video calls in recent years proved to be as effective because it is the closest method that creates an in-person experience while being geographically separate [33]. Additionally, it appears that in-person interviews are slightly superior to those carried out using video calls as interviewees tend to say more when interviewed in person, but the difference is small. Therefore, certain constraints such as difficulties in travelling because of the pandemic may justify the use of an alternative and effective data-collection method such as video calls [33].

## Conclusion

Overlapping and interlinked crises in Lebanon have contributed to widespread deprivation among Syrian refugees and increased economic concerns about the future. Also, the crises may have influenced fertility preferences and SRH behaviours: women report wanting to limit family size and seeking modern and long-acting contraceptives to facilitate that choice. Interestingly, the crises appear to have shifted power within households as women were forced to work and men struggled to maintain employment sufficient to support families. This resulted in women reporting less pressure to have (additional) children and less anxiety about husbands seeking to remarry.

## Data Availability

The datasets used and/or analysed during the current study are available from the corresponding author on reasonable request.

## Abbreviations

Covid-19: Corona Virus
FGDs: Focus group discussions
FP: Family planning
ITS: Informal tented settlements
LBP: Lebanese pound
NA: Not applicable
RHS: Reproductive health services
SRH: Sexual and reproductive health
SRHS: Sexual and reproductive health services
SMEB: The survival and minimum expenditure baskets
UNHCR: The United Nations High Commissioner for Refugees
VASYR: The vulnerability assessment of Syrian refugees

## Footnotes

1 A reference that allows humanitarian actors in Lebanon to assess the components and volume of purchasing conducted by Syrian refugee households to meet their basic needs.
